# Nitrogen and Potassium Concentrations in the Nutrients Solution for Melon Plants Growing in Coconut Fiber without Drainage

**DOI:** 10.1155/2013/546594

**Published:** 2013-06-23

**Authors:** Luiz Augusto Gratieri, Arthur Bernardes Cecílio Filho, José Carlos Barbosa, Luiz Carlos Pavani

**Affiliations:** ^1^Instituto Federal de Educação, Ciência e Tecnologia Sul de Minas Gerais (IFSULDEMINAS), Campus Muzambinho, Morro Preto s/n, 37890-000 Muzambinho, MG, Brazil; ^2^Universidade Estadual Paulista, Via de Acesso Prof. Paulo D. Castellane s/n, 14884-900 Jaboticabal, SP, Brazil

## Abstract

With the objective of evaluating the effects of N and K concentrations for melon plants, an experiment was carried out from July 1, 2011 to January 3, 2012 in Muzambinho city, Minas Gerais State, Brazil. The “Bonus no. 2” was cultivated at the spacing of 1.1 × 0.4. The experimental design was a randomized complete block with three replications in a 4 × 4 factorial scheme with four N concentrations (8, 12, 16, and 20 mmol L^−1^) and four K concentrations (4, 6, 8, and 10 mmol L^−1^). The experimental plot constituted of eight plants. It was observed that the leaf levels of N and K, of N-NO_3_ and of K, and the electrical conductivity (CE) of the substrate increased with the increment of N and K in the nutrients' solution. Substratum pH, in general, was reduced with increments in N concentration and increased with increasing K concentrations in the nutrients' solution. Leaf area increased with increments in N concentration in the nutrients solution. Fertigation with solutions stronger in N (20 mmol L^−1^) and K (10 mmol L^−1^) resulted in higher masses for the first (968 g) and the second (951 g) fruits and crop yield (4,425 gm^−2^).

## 1. Introduction

The fruit of the melon plant is highly appreciated all over the world. In 2010, among fruits in general, melon was the first item of the Brazilian exportations. According to Agrianual [1] data, 177,829 tons of melons were exported. The main melon producing area is in the semiarid region of the northeast of Brazil which supplies all the melons for the internal market. Simultaneously in the last few years, the production of noble melons (*Cucumis melo *L., *cantalupensis *group) is growing steadily in the southeast region, already representing 15 to 20% of the market [2].

In the Southeast region, melon is cultivated mainly under protected conditions due to pluviosity in that region being frequently very high. The cultivation of net melon over the soil surface has met several phytosanitary problems so that alternative cultivation procedures have been sought. One of the most important ones is that melon plants are cultivated in a substratum which, if properly managed, permits yields superior to those of the cultivation on soil [3].

Nowadays, cultivating in substrata has its management founded on fertigation and drainage of a certain percentage of the applied volume in order to keep substratum conditions adequate for the crop [4, 5]. On the other hand, this management causes a high residual volume, not used by the plants, which, when discarded, can contaminate the soil and water fountainheads [6, 7]. In addition to that, it is necessary to consider the direct influence on the production costs since what is being discarded is a part of the nutrients solution [8]. So, this management is to be used when the water for the preparation of the nutrients solution has a high electric conductivity. In Brazil, in the majority of the agricultural regions, the water is of excellent quality. So, the challenge to be overcome is the development of a management for the cultivation in coconut fiber in order to maintain the nutrients solution concentrations and the substratum chemical attributes at adequate levels. The lack or excess of the nutrients solution may affect the growth and the productivity of the plants [9].

K and N are the macronutrients that melon plants extract more—N, approximately 38% and K, approximately 45% of total of nutrients [10]. On the other hand, according to information in the literature, the concentrations of those two nutrients in the nutrients solution vary in function of cultivar, substratum, the climatic conditions, the forms and frequency with which water and nutrients are supplied, and the plant physiological stage among other factors which have influence on the growth and the mineral composition of the plant [11–13]. Studies concerning aspects of the nutrients solution in which melon plants are cultivated in coconut fiber without drainage were not found. Since there is no defined criterion for the use of fertigation in that system, the recommended N and K concentrations, when drainage is part of the management system, may not be the adequate concentrations when drainage is not part of that system.

So, the objective of this investigation was to evaluate the most efficient N and K concentrations in the nutrients solution when melon plants are cultivated in coconut fiber without drainage.

## 2. Materials and Methods

The experiment was carried out from July 1, 2011 to January 3, 2012, at the South Federal Institute of Minas Gerais, in Muzambinho (South latitude of 21°22′33′′, West longitude of 46°31′33′′, and at a mean altitude of 1000.75 m above sea level), in Muzambinho, Minas Gerais State, Brazil. The minimum, maximum, and mean temperatures during the experimental period were, respectively, 15.5, 35.6, and 25.0°C. A 30 × 14 m, 4 m high greenhouse, covered with a low density, 150 *μ*m thick polyethylene film, with lateral and frontal shutting up to 3 m with a polypropylene screen which resulted in a shading of 30% was used.

Four N concentrations (N_1_ = 8, N_2_ = 12, N_3_ = 16, and N_4_ = 20 mmol L^−1^) and four K concentrations (K_1_ = 4, K_2_ = 6, K_3_ = 8, and K_4_ = 10 mmol L^−1^) were used; this resulted in 16 treatment combinations. These treatment combinations, each repeated 3 times, were arranged in the greenhouse according to a randomized complete block design. The melon genotype used in this experiment was the “Bonus no. 2,” a F_1_ hybrid belonging to the *reticulatus *botanical variety of the “net” type. 

Seed sowing took place on September 21, 2011 in 128-celled expanded polystyrene trays, these cells being filled with coconut fiber powder. The trays were daily fertigated till the appearing of the first noncotyledonary leaf. The nutrients solution for this period of fertigation had half the concentrations of nutrients.

Fifteen days after sowing (DAS), the seedlings were transplanted to the cultivation channels at the spacing of 1.10 m between lines and 0.40 m between plants. Each channel was 0.2 m wide, 0.19 m high, and 3 m long. The channel was covered with a double-face polyethylene film and filled with 17.2 kg of the Golden-Mix 80 coconut fiber substratum, this amount meaning 0.01425 dm^−3^ per plant. This substratum results from a fifty-fifty mixture of coarse-texture and fine-texture substrata. The substratum on which the plants grew had the following chemical features: pH = 6.7, EC = 0.2 dS m^−1^, and, in mg L^−1^, 0.3 of N-NO_3_^−^, 1.2 of N-NH_4_^+^, 24.8 of K, 2.3 of P, 0.4 of Ca, 0.1 of Mg, and 0.03 of Zn. The maximum water retention capacity displayed by the substratum was 356 mL L^−1^.

The substratum top was covered with a polyethylene film with the white side upwards. The cultivation channels were placed at the same level on top of the terrain with their extremities closed by the same film. The plants, supported by stalks, grew vertically. The basal secondary branches were pruned up to the tenth node, and, after that, the plants were allowed to grow freely to support the fruits. The pollination was carried out by bees with free access to the plants until there were four fruits per plant. After that, the plant buds were eliminated and two fruits suppressed so that there were two fruits per plant, one at the 11th node and the other at the 13th node. The apical buds of secondary branches, located after the third leaf, were eliminated and, if on the main branch, after the 22nd node. The first and the second fruits borne by each plant were harvested 83 and 89 days after transplantation (DAT), respectively. Preventive and curative measures were taken for phytosanitary reasons.

A drip irrigation system was adopted by the use of antidrainage and self-compensating emitters placed 0.4 m one from another with a uniformity of 99% and a water flow of 4.5 L h^−1^ under the operating conditions. 

The water used to prepare the nutrients solution had the following characteristics: pH = 6.6 and, in mg L^−1^, 0.47 of Zn, 0.0067 of Cu, 6.8 of chlorides, 0.30 of Fe, 0.0004 of N-NO_2_^−^, 0.5 of N-NO_3_^−^, 0.0019 of N-NH_4_^+^, 14.6 of S, 1.4 of B, and absence of chlorine, organic N, phosphorus, orthophosphate, potassium, sodium, calcium, and magnesium. 

The characteristic curve of water retention by the substratum was determined for water tensions in the substratum up to 100 kPa. Fertigation was controlled by sensors of the Irrigas matricial tension type to be used in substrata capable of measuring tensions between 0 and 15 kPa. Fertigation was started soon after the plants were transplanted up to harvest with a frequency which was determined by the climatic conditions and the melon plant phonological stage. The irrigation duration was of 3 minutes and the substratum moisture tension to initiate fertigation was variable during the plant cycle (Table 1).

N and K levels in the leaves were evaluated making use of the 5th leaf starting from the tip of the branch, excluding the apical tuft, at the beginning of fruit set, which took place 46 DAT [14]; 60 DAT the pH (pH meter DIGIMED—model DM 21), the electrical conductivity (TECNAL—model TEC 4MP conductivimeter), the concentrations of K (Compaction ion meter C-131 Horiba Cardy), and N (distillation method) were also determined. In order to make those measurements, four samples per plot, at a distance of 0.10 m from the plant stem, between 6:30 and 7:30 a.m., were taken. These samples were mixed to make a composed sample. The analyses were made using the 1 : 1.5 extraction method [15]. Leaf area was measured at the end of the cycle (89 DAT) by measuring the width of all leaves of the plant and making use of the mathematical model (AF = 0.826 L^1.89^ (*R* ^2^ = 0.97)) to calculate it [16]. First and second fruits had their weight determined and also total yield in kilograms of fruits per plant. 

The data were submitted to the analysis of variance by the *F* test and the polynomial regression analysis. The data related to the first and second fruits weight and the total yield per plant were analyzed by a regression study by the response surface methodology analysis. 

## 3. Results and Discussion 

**Figure 1:**
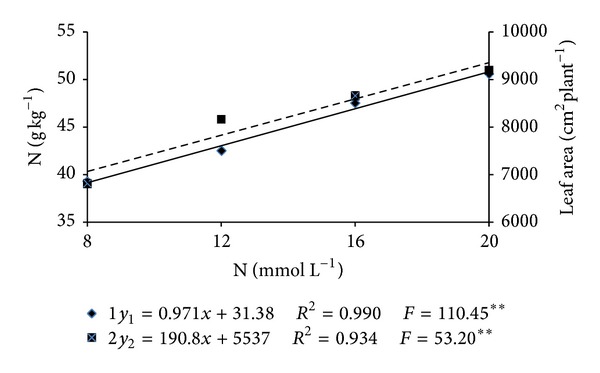
N content in the leaf used for the evaluation of the plant nutritional status 46 days after transplantation (DAT) (Y1) and leaf area 89 DAT (Y2) as influenced by the concentrations of N in the nutrients solution.

Measurement made 46 DAT showed that leaf N content was significantly influenced only by the N concentration in the nutrients solution (Table 2) with the means showing adjustment to a first-degree equation (Figure 1). An increment of 30% in the N level (39.1 and 50.8 g kg^−1^ of N) was verified when the nutrients solution used to fertigate the plants had the smallest and the highest N concentration, respectively.

**Figure 2:**
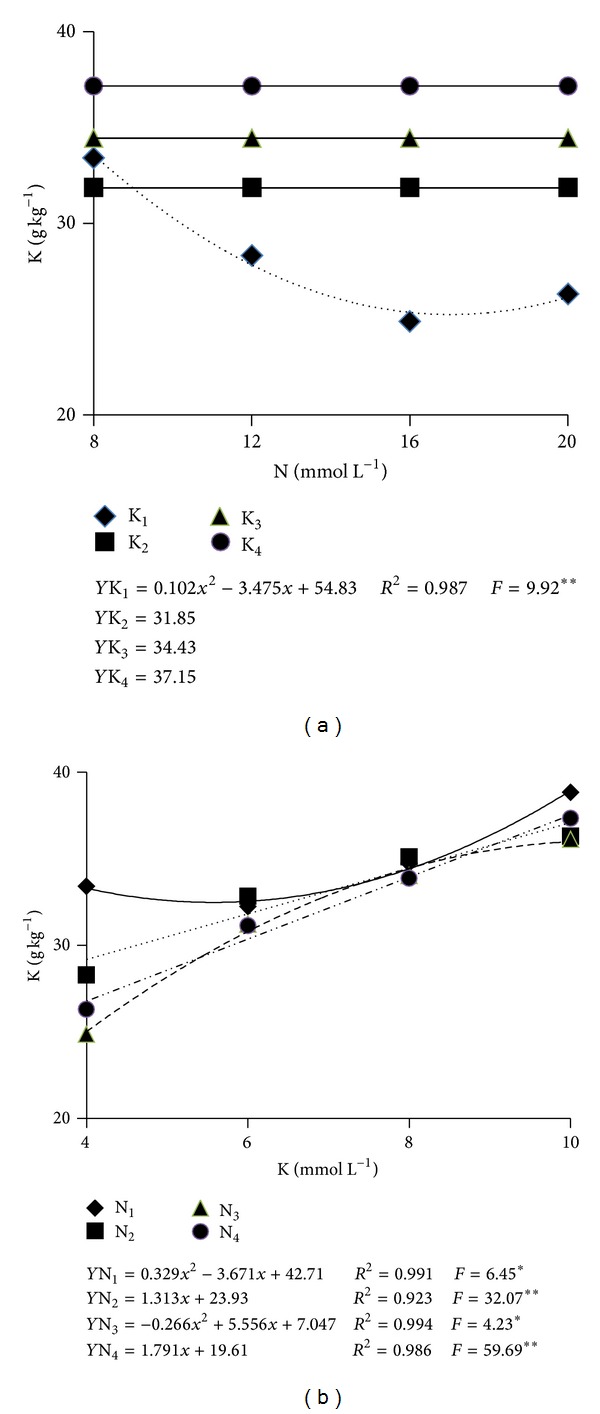
K content in the leaf used for the evaluation of the nutritional status of “Bonus no. 2” melon plants as influenced by N (a) and K (b) concentrations in the nutrients solution. **, *: Significant at the levels of 1 and 5%, respectively, according to the *F* test.

The K leaf content was influenced by the interaction of the factors (Table 2). Significant adjustments to first- and second-degree equations were verified in accordance with the N and K combination (Figure 2). While in the nutrients solution of 4 mmol L^−1^ of K the increase in N concentration resulted in a reduction in the level of K in the leaves. In the concentrations of 6, 8, and 10 mmol L^−1^ of K, no significant adjustment to a polynomial equation was observed and resulted in the levels of 31.9, 34.4, and 37.2 g kg^−1^ of K in the leaves, respectively (Figure 2(a)). On the other hand, in all N concentrations increments in K concentration were verified with each increment in K concentration, and the highest levels were observed when the nutrients solution had the lowest concentration of N (Figure 2(b)), this being explained by the lower growth of leaf area with the lowest concentrations of N (Figure 1).

Among the solutions with the lowest and the highest concentrations of K in the nutrients solution (4 and 10 mmol L^−1^), an increment of 55.6% in the K leaf content (25.0 and 38.9 g kg^−1^) was verified. Even with all the variation observed in the levels of K and N resulting from the different concentrations of these elements in the nutrients solution, all the leaf levels of N and K were verified to be within the range of values considered as adequate of 15 to 50 g kg^−1^ for N and of 25 to 40 g kg^−1^ for K [14, 17].

**Figure 3:**
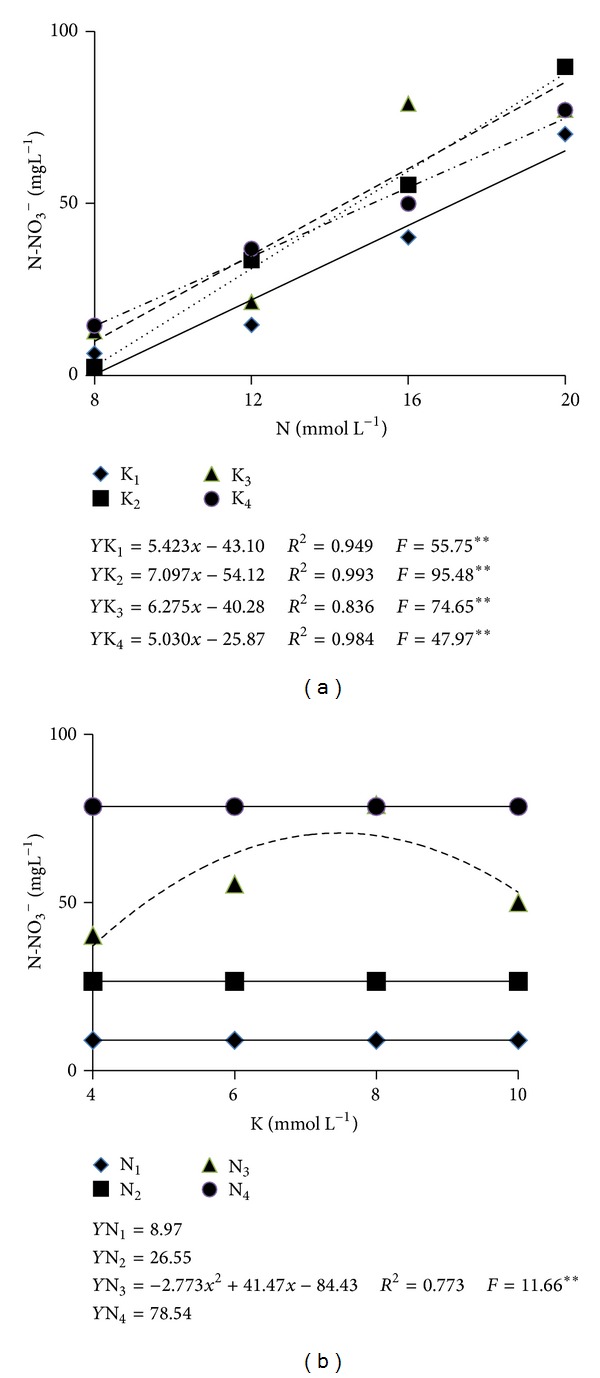
N-NO_3_^−^ levels (mg L^−1^) in the substratum solution extract (method 1 : 1.5 v/v) 60 days after transplantation as influenced by the concentrations of N (a) and of K (b) in the nutrients solution. **: Significant at the level of 1%, according to the *F* test.

The N-NO_3_^−^ level in the substratum was significantly influenced by the interaction between the concentrations of N and K (Table 2). For all K concentrations, the higher the concentration of N in the nutrients solution, the higher the level of N-NO_3_^−^ in the substratum. The lowest level, that is, 0.28 mg L^−1^, resulted from the lowest concentrations of N and K whereas the highest, that is, 87.8 mg L^−1^, resulted from 20 of N and 6 mmol L^−1^ of K (Figure 3(a)). On the other hand, the breaking down of the interaction degrees of freedom for the level of N-NO_3_^−^ as a function of the N concentration in each K concentration, showed that a significant adjustment was verified only for the nutrients solution containing 16 mmol L^−1^ of N with the maximum level (70 g L^−1^) resulting from 7.5 mmol L^−1^ of K. In the solutions with 8, 12, and 20 mmol L^−1^ of N, nitrate means in the substratum were 9.0, 26.6, and 78.5 mg L^−1^, respectively (Figure 3(b)). The range from 4 to 6 mmol L^−1^ or 56 to 84 mg L^−1^ is adequate for the majority of crop species growing in organic substrata [18]. It is, thus, observed that only the nutrients solution with at least 16 mmol L^−1^ of N could result in N-NO_3_^−^ levels in the substratum within the range mentioned by that author. 

The increment in N-NO_3_^−^ availability in the substratum may be explained by the concentration of N in the nutrients solution and by the demand of the nutrient by melon plants. The levels of N-NO_3_^−^ in the substratum solution extract were low when N and K concentrations were at their lowest (8 and 4 mmol L^−1^). In this case, since the N concentration in the nutrients solution is low, the N added to the substratum via fertigation was used by the plant in almost its totality, this resulting in the lowest availability of N-NO_3_^−^ in the substratum. This same observation was reported by Gaion et al. [19] between 45 and 78 DAT, when the solution used had a lower concentration of N (12 mmol L^−1^), and the reduction was attributed to the high N demand by fructifying melon plants.

**Figure 4:**
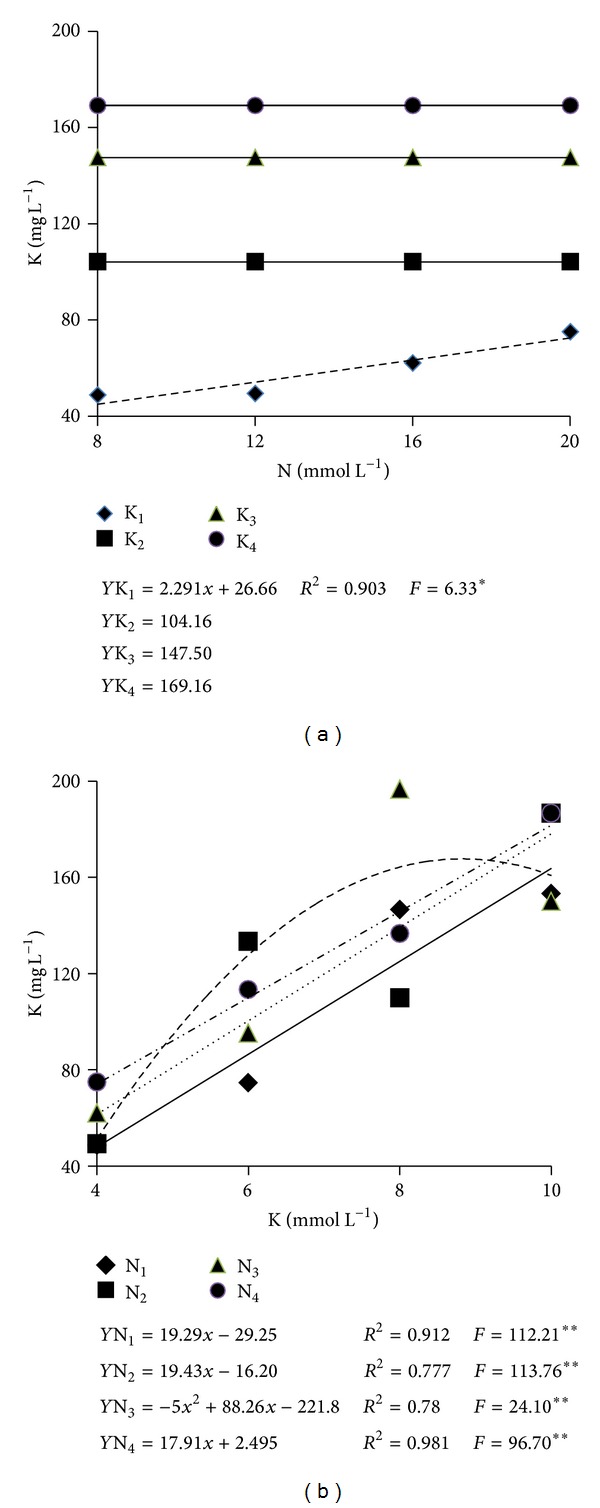
K levels (mg L^−1^) in the substratum solution extract (method 1 : 1.5 v/v) 60 days after transplantation as influenced by the concentrations of N (a) and of K (b) in the nutrients solution. **, *: Significant at the levels of 1 and 5%, respectively, according to the *F* test.

The substratum K content was significantly influenced by the interaction between N and K concentrations (Table 2). The level of K as influenced by the N concentration only showed a significant equation adjustment for the solution with 4 mmol L^−1^ of K in which the K level in the substratum increases linearly with the increment in the concentration of N, reaching a maximum of 72.5 mg L^−1^. In the nutrients solutions in which the K concentrations were of 6, 8, and 10 mmol L^−1^, the mean K concentrations in the substratum were of 104.2, 147.5, and 169.2 mg L^−1^, respectively (Figure 4(a)). This increment in K level in the substratum is observed in the significant adjustments of equations for each N concentration as influenced by K concentrations in the nutrients solution (Figure 4(b)).

The highest level of K in the substratum (181.6 mg L^−1^) was observed with fertigation of the nutrients solution which had the highest concentrations of N and 10 mmol L^−1^ of K whereas the solution with 4 mmol L^−1^ of K resulted in K level in the substratum solution extract lower than the range considered as adequate by Baumgarten [18]. According to that author, for the majority of the crop species, the ideal range of K is between 1.9 and 3.5 mmol L^−1^ or 74.3 and 136.8 mg L^−1^ when the dilution extraction method 1 : 1.5 v/v is used. On the other hand, when the solutions with 8 and 10 mmol L^−1^ of K are used, the levels found in the substratum solution extract are higher than those of the optimum range.

**Figure 5:**
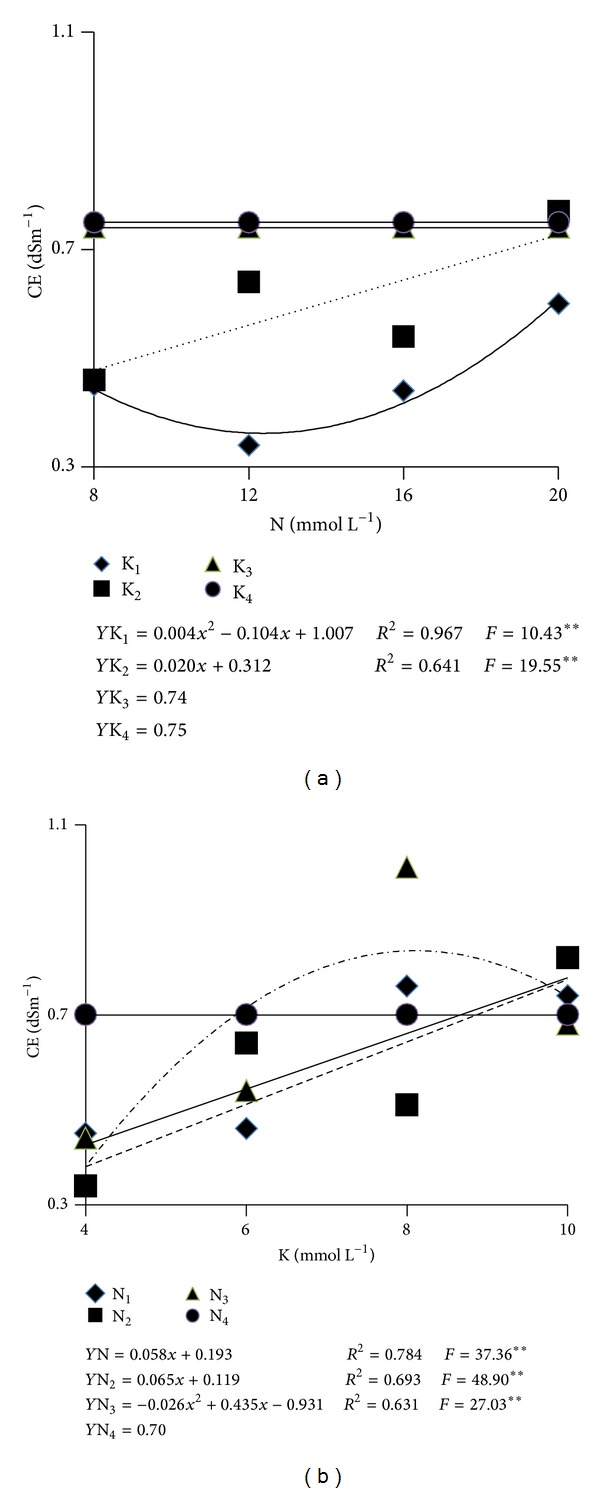
Electrical conductivity of the substratum solution extract (method 1 : 1.5 v/v) 60 days after transplantation as influenced by the concentrations of N (a) and K (b) in the nutrients solution. **: Significant at the level of 1%, according to the *F* test.

The substratum electrical conductivity (CE) was significantly influenced by the interaction of the concentrations of N and K (Table 2). The mean CE values for the substratum fertigated with solutions with 8 and 10 mmol L^−1^ of K did not adjust to polynomial equations in response to the increments in N concentrations and showed very similar results—0.74 and 0.75 dS m^−1^. Different from that, when the substratum was fertigated with nutrients solution with the lowest concentrations of K, an increment in the extract CE was verified as the concentrations of N increased (Figure 5(a)).

When the substratum solution extract CE study was undertaken as influenced by increments in K concentration in the nutrients solution, polynomial equations were not verified to describe the observed effect only when the N concentration was the highest (20 mmol L^−1^) whereas the responses to the nutrients solutions with 8 and 12 mmol L^−1^ adjusted themselves linearly to increments of K in the nutrients solution. The solution with 16 mmol L^−1^ of N resulted in the highest substratum CE when containing 8 mmol L^−1^ (Figure 5(b)). Therefore, between the nutrients solution with higher or lower concentrations of N and K, the substratum solution extract saline index underwent a more than twofold increment—it went from 0.33 to 0.89 dS m^−1^ (Figure 5(b)). This increment can be partially attributed to the augments in the levels of nitrate and potassium in the substratum. 

Only when nutrients solutions containing at least 8 mmol L^−1^ of K, independently of the N concentration, or solutions with 16 mmol L^−1^ of N and a minimum of 6 mmol L^−1^ of K or 20 mmol L^−1^ of N and any K concentration, the substratum CE reached values close to the inferior limit of the range considered adequate by Baumgarten [18], that is, from 0.8 to 1.5 dS m^−1^ and by Cavins et al. [20], that is, from 0.76 to 1.5 dS m^−1^ as determined by the extraction methods by dilution 1 : 1,5 v/v and 1.2 v/v, respectively.

Nutrients solutions with concentrations of N and K lower than those mentioned previously resulted in very low substratum solution extract CE values. Therefore, the adopted management of closed channels without drainage of part of the nutrients solution applied for the lixiviation of nutrients, did not cause the feared substratum salinization probably due to the water quality, which was very poor in ions. 

**Figure 6:**
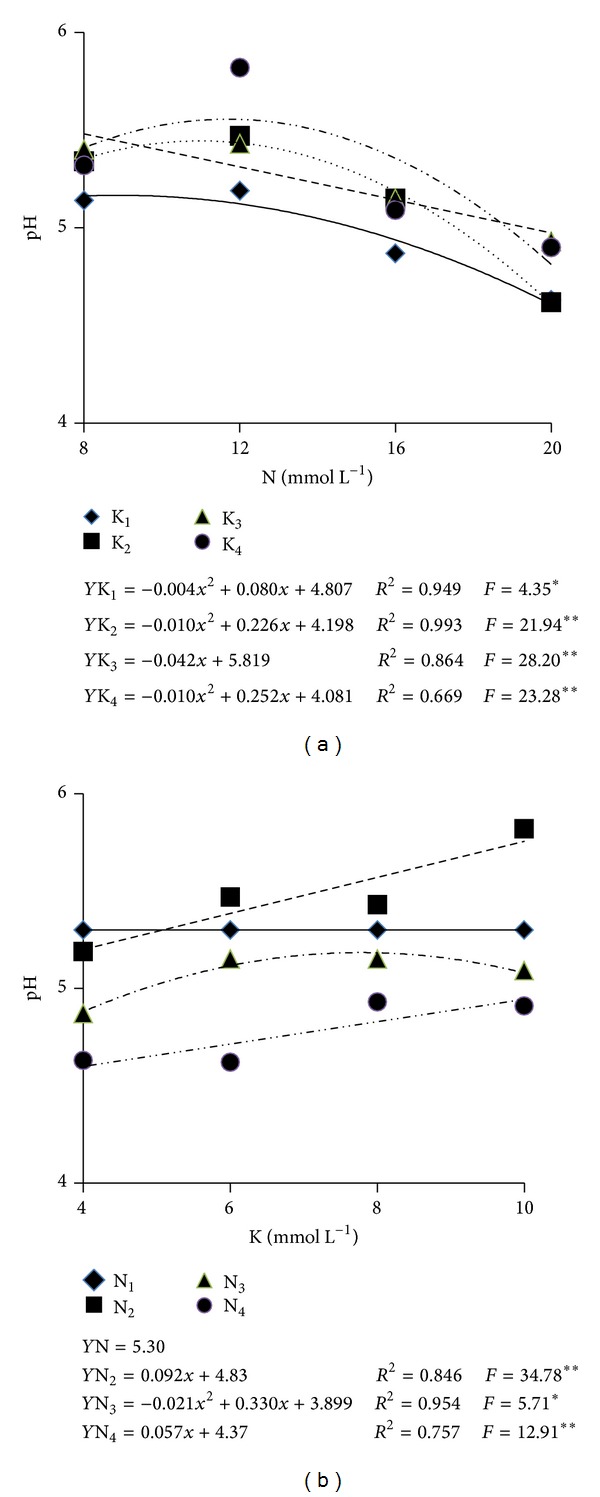
pH values of the substratum solution extract (method 1 : 1.5 v/v) 60 days after transplantation as influenced by the concentrations of N (a) and K (b) in the nutrients solution. **, *: Significant at the levels of 1 and 5%, respectively, according to the *F* test.

The substratum pH was significantly influenced by the interaction between N and K concentrations (Table 2). For each K concentration in the nutrients solution as N concentration increased, there was a reduction in pH (Figure 6(a)).

In the study concerning pH values as influenced by augments in K for each N concentration, increments in pH values were verified with increasing K concentrations (Figure 6(b)). The lowest pH value was 4.7, which resulted from the fertigation with a solution containing 20 and 6 mmol L^−1^ of N and K, respectively. The highest pH value was 5.7, which resulted from nutrients solution containing 12.6 and 10 mmol L^−1^ of N and K, respectively. 

It is probable that the augment in pH was a consequence of the fertilizers used to prepare the formulations. In the nutrients solution containing 8 and 12 mmol L^−1^ of N, the source of K was potassium chloride whereas in the 16 and 20 mmol L^−1^ of N, the K source was potassium nitrate. It is possible that the higher chloride concentration in the solutions with the lowest amount of N may have induced a larger absorption of this anion and, therefore, an increment in hydroxide (OH^−^) concentration in the substratum, and in raising pH values [21]. According to data published by Martinez [22], the substratum pH values increase when the plants are in high demand of nitrate. In this case, however, the ion nitrate was of the same concentration in all the nutrients solutions.

N concentrations above 12 mmol L^−1^ caused the pH to decrease and reach the lowest value when the highest N concentration was used (20 mmol L^−1^). pH values were 4.8, 4.7, 5.0, and 5.1 for the K concentrations of 4, 6, 8, and 10 mmol L^−1^, respectively. Gaion et al. [19] reported pH values lowering from 6.3 to 5.0 during melon plants cycle in which the plants grew in a mixture of sand and peanut shell. Such reduction may be due to the plant extracting cations of basic nature (Ca, Mg, K, and Na), and this resulted in the increment of H^+^ [23] or to the excessive use of ammoniacal fertilizers. On the other hand, this hypothesis is not applicable to this work since the concentration of N-NH_4_^+^ in the nutrients solution did not reach values above 10%. On the other hand, the first hypothesis is more likely to explain the results at 38 DAT the first feminine flowers sprouted and at 41 DAT fructification started, and this caused an increment in nutrients demand by the plants, specially for potassium as reported by Lester et al. [24]. As a consequence, the roots excrete more H^+^, thus reducing pH. 

The pH values observed in this work are very close to the pH range recommended by several authors for organic substrata: 6.19 [18], 5.7 to 6.0 [23], 5.4 to 6.0 [25], 5.8 to 6.2 [26], and 5.4 to 6.4 [27]. Notwithstanding, the lowest value found (4.7) is below the values recommended by those authors. pH values were not measured at the end of the cycle, and the analysis of the result at 60 DAT shows that the value at 89 DAT, when harvesting was made, may have reached critical values.

Melon plants leaf area (AF) was affected only by N concentration (Table 3). Leaf area measurements adjusted to first-degree equations and increased linearly with N concentration in the nutrients solution (Figure 1). Leaf areas resulting from N concentrations of 8 and 20 mmol L^−1^ in the nutrients solution were of 7,063 and 9,353 cm^2^ per plant, respectively. The expressive effect of N on melon plant growth has been intensively reported in the literature [13, 28, 29]. The highest melon plants AF were verified when the highest N and N-NO_3_^−^  leaf levels were found in the substratum solution extract (Figure 2(a)).

The mass of the first melon fruit (MPF) was significantly affected by N concentration whereas the mass of the second fruit (MSF) and productivity (*P*) were influenced not only by N concentration but also by K concentration (Table 3).

**Figure 7:**
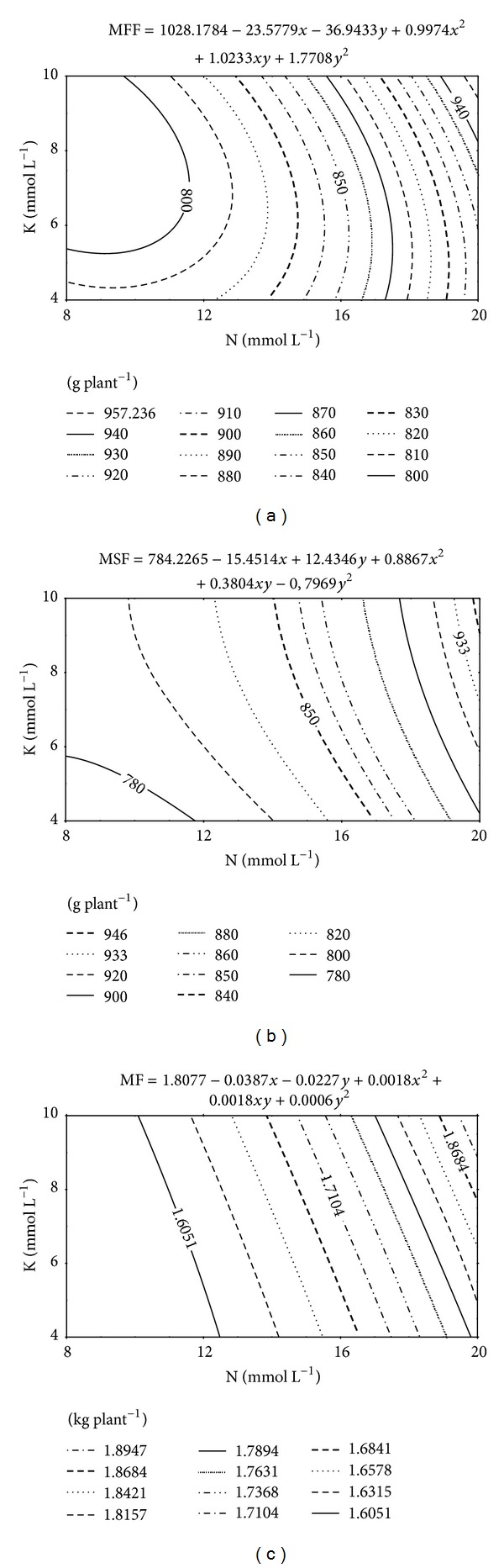
Mass of the first fruit (MFF) (a), of the second fruit (MSF) (b), and of fruits (MF) (c) as influenced by the concentrations of N and K in the nutrients solution.

According to a response surface methodology analysis, the lowest MPF (817 g, according to Figure 7(a)) and MSF (766 g, as shown in Figure 7(b)) values were verified when the melon plants were fertigated with a nutrients solution with the lowest concentrations of N and K (8 and 4 mmol L^−1^). As the concentrations of N and K were increased, higher MPF and MSF were attained. MPF and MSF increased by 18.5 and 24.0% reaching 968 and 951 g, respectively.

Productivity (Figure 7(c)) mirrored what happened with MPF and MSF. An increment in productivity of 22.5% (total fruit weight went from 1,589 g to 1,947 g per plant, or, 3.611 kg to 4.425 kg m^−2^) was observed when the solutions with the lowest and the highest concentrations in N and K were used.

Considering the K concentration of 10 mmol L^−1^, which was the highest of this element, the increment in N concentration from 8 to 20 mmol L^−1^ resulted in an increment of 356 g in productivity, that is, 29.7 g for each 1 mmol L^−1^ of N added to the nutrients solution. 

On the other hand, keeping the concentration of 20 mmol L^−1^ of N and increasing the K concentration from 4 to 10 mmol L^−1^, the increment was of 131 g, or, 21.8 g for each mmol L^−1^ of K added to the nutrients solution. This analysis makes clear that the effect of N on fruit productivity is larger than that of K. This is probably due to the effect N has on plant leaf area. The larger productivity did also correlate with the largest N and K leaf contents as well as with the levels of these nutrients in the substratum solution extract. 

Therefore, higher concentrations of N and K in the nutrients solution in the interval from 8 to 20 mmol L^−1^ of N and from 4 to 10 of K cause increased levels of these nutrients in the plant leaves and in the substratum, of electrical conductivity, of leaf area, fruit mass, and productivity.

## Figures and Tables

**Table 1:** Plant cycle in days after transplantation (DAT), maximum water tension in the substratum (MWT), and nutrients solution volume (SNV), as influenced by N concentration in the nutrients solution.

Cycle (DAT)	MWT (kPa)	SNV (L)
0–12	1.0	3.12
13–33	1.0	6.60
34–52	2.0	23.18
53–59	4.0	11.07
60–89	5.0	31.96

		Total = 75.95

**Table 2:** Analysis of variance results for nitrogen leaf level (LN) and potassium leaf level (LK), N-NO_3_^−^, hydrogen ion potential (pH), electrical conductivity (CE), and N (NS) and K (KS) concentrations in the solution of substratum.

Treatments	LN	LK	pH	CE	NS	KS
	(g kg^−1^)		(dS m^−1^)	(mg L^−1^)	
N						
N_1_	39.31	34.80	5.30	0.60	8.97	105.83
N_2_	42.51	33.13	5.48	0.58	26.55	119.83
N_3_	47.52	31.57	5.07	0.67	56.12	126.00
N_4_	50.59	32.16	4.77	0.70	78.54	127.92

*F *	37.17**	7.43**	74.05**	7.29**	90.44**	6.01**

K				
K_1_	45.12	28.22	4.96	0.46	32.82	58.75
K_2_	45.03	31.86	5.14	0.60	45.24	104.17
K_3_	44.12	34.43	5.23	0.74	47.57	147.50
K_4_	45.67	37.15	5.29	0.75	44.56	169.17

*F *	0.60^NS^	53.84**	16.44**	42.15**	4.15*	144.11**

N × K	0.93^NS^	2.63*	2.99*	11.86**	2.39*	10.58**

C.V. (%)	6.37	5.46	2.39	11.50	26.45	11.77

**, *, NS: Significant at the levels of 1 and 5% and non significant, respectively, according to the *F* test.

**Table 3:** Analysis of variance results for leaf area (LA), mass of the first fruit (FFM), mass of the second fruit (SFM), and fruit productivity per plant (*P*) as influenced by the concentrations of N and K in the nutrients solution.

Treatments	LA	FFM	SFM	*P *
	(cm^2^ plant^−1^)	(g)	(g)	(g)
N				
N_1_	309.86	789.83	776.58	1568.33
N_2_	371.11	835.50	820.75	1657.50
N_3_	393.78	834.25	832.33	1668.33
N_4_	417.99	943.75	933.25	1870.00

*F *	18.98**	39.33**	33.54**	47.01**

K				
K_1_	379.56	847.33	813.00	1654.16
K_2_	367.26	853.75	851.50	1705.00
K_3_	374.83	833.75	836.33	1672.50
K_4_	371.09	868.50	862.08	1732.50

*F *	0.24^NS^	1.91^NS^	3.47*	3.49*

N × K	0.57^NS^	1.96^NS^	1.34^NS^	1.92^NS^

C.V. (%)	9.87	4.25	4.71	3.81

**, *, NS: Significant at the levels of 1 and 5% and non significant, respectively, according to the *F* test.
